# Familial/inherited cancer syndrome: a focus on the highly consanguineous Arab population

**DOI:** 10.1038/s41525-019-0110-y

**Published:** 2020-02-03

**Authors:** Fawz S. AlHarthi, Alya Qari, Alaa Edress, Malak Abedalthagafi

**Affiliations:** 1 Genomics Research Department, Saudi Human Genome Project, King Fahad Medical City and King Abdulaziz City for Science and Technology, Riyadh, Saudi Arabia; 2 Genetics Counselling Division, Saudi Diagnostic Laboratory, King Faisal Specialist Hospital International Company, Riyadh, Saudi Arabia; 3 Medical Genetic Department, King Faisal Specialist Hospital and Research Centre, Riyadh, Saudi Arabia

**Keywords:** Genetics, Cancer

## Abstract

The study of hereditary cancer, which accounts for ~10% of cancer cases worldwide is an important subfield of oncology. Our understanding of hereditary cancers has greatly advanced with recent advances in sequencing technology, but as with any genetic trait, gene frequencies of cancer-associated mutations vary across populations, and most studies that have located hereditary cancer genes have been conducted on European or Asian populations. There is an urgent need to trace hereditary cancer genes across the Arab world. Hereditary disease is particularly prevalent among members of consanguineous populations, and consanguineous marriages are particularly common in the Arab world. There are also cultural and educational idiosyncrasies that differentiate Arab populations from other more thoroughly studied groups with respect to cancer awareness and treatment. Therefore, a review of the literature on hereditary cancers in this understudied population was undertaken. We report that *BRCA* mutations are not as prevalent among Arab breast cancer patients as they are among other ethnic groups, and therefore, other genes may play a more important role. A wide variety of germline inherited mutations that are associated with cancer are discussed, with particular attention to breast, ovarian, colorectal, prostate, and brain cancers. Finally, we describe the state of the profession of familial cancer genetic counselling in the Arab world, and the clinics and societies dedicated to its advances. We describe the complexities of genetic counselling that are specific to the Arab world. Understanding hereditary cancer is heavily dependent on understanding population-specific variations in cancer-associated gene frequencies.

## Introduction

Cancer remains a leading cause of morbidity and mortality across the globe,^1,2^ with increases in mortality of approximately 25.0% since the 1990s and projections of ≥23 million cases annually by 2030.^3,4^ Hereditary causes account for ~10% of cancer cases, and an estimated 20% of cancer patients have a positive family history of cancer.^5–7^

Hereditary cancer syndrome is defined as an elevated risk of cancer that runs in the family. The risk originates from heritable mutations in specific genes.^8,9^ The type of cancer is dependent on the mutated gene. Hereditary breast and ovarian cancers originate from *BRCA1* and/or *BRCA2* gene mutations that significantly increase the likelihood of developing breast, ovarian, prostate and other types of cancer.^1,10^ Patients with Li-Fraumeni syndrome, characterised by *TP53* mutations, have a heightened risk of cancer before age 30, and are almost guaranteed to suffer from cancer by the age of 60.^8,11–13^ Carriers of cancer syndrome associated genes also have a higher risk of multiple malignancies and rare cancers, and are more likely to develop cancer at a younger age.

Advances in sequencing technologies, particularly, high throughput sequencing have permitted the discovery of novel genes responsible for cancer heritability, facilitating efficient genetic screening.^14–16^ The major genetic changes in cancer include single nucleotide variants (SNVs); duplications, insertions, or deletions; exon and gene copy number changes; and structural variants (SVs).^17^ The molecular profiling of heritable cancer genes ranges from simple assessments of known hotspot mutations in single genes, to more complex tests that simultaneously detect all gene alterations using allele-specific PCR, Sanger sequencing, multiplex ligation-dependent probe amplification (MLPA), pyrosequencing or mass spectrometry (MS).^17,18^ Gene copy numbers and SVs can also be assessed through fluorescence in situ hybridisation (FISH). Next generation sequencing (NGS) technologies have revolutionised molecular profiling permitting whole exome sequencing (WES) that examines all protein-coding regions and whole genome sequencing (WGS) that profiles protein-coding and non-coding regions. Example NGS technologies include Illumina MiSeq and HiSeq and the Life Technologies Ion Torrent personal genome machine.^17–19^ These technologies can permit the identification of a family history of cancer and help identify those at-risk and likely to benefit from enhanced surveillance and early detection. Patients diagnosed with cancer syndromes do not necessarily develop cancer, but awareness of their status may enable early detection to prevent mortality.^20,21^ Accordingly, increased public awareness that cancer can be heritable, and that the heritable risk can be evaluated has increased as has the demand for genetic counselling and screening.^14,22–24^

**Fig. 1:**
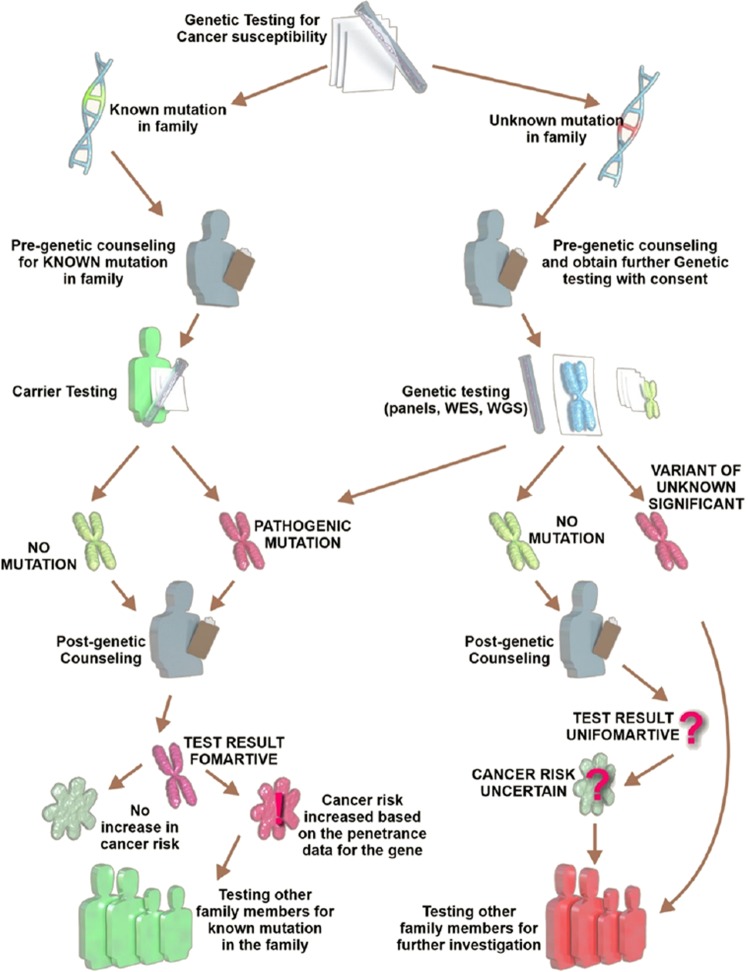
Genetic counselling process for cancer susceptibility.

The incidence and prevalence of hereditary cancer amongst different ethnic populations is often distinct. Cancer is a major problem in the Arab world^4,25–31^ which is delimited by Lebanon and Syria to the north, Morocco to the west, south to Yemen, and Iraq in the east, accounting for >300 million people. The incidence of cancer in Arab countries has increased over the last 10 years, primarily due to lifestyle changes and obesity, as traditional foods are replaced with Western-diets.^25^ For example, SA, Qatar, Kuwait, UAE, Bahrain, and Saudi Arabia are amongst the top ten countries for obesity prevalence, which for breast cancer alone has increased the incidence rates by ~2% in adult males and ~7% in adult females.^32^ Barriers to cancer screening in addition to a lack of cancer education remain problems in the region.^31^ From the perspective of hereditary cancer, genetic disorders occur at a high frequency in several Arab communities due to high rates of inbreeding, with 25–60% of all marriages being consanguineous, with common first cousin marriages. Problems are compounded by the lack of public health measures directed towards the prevention of congenital and genetic disorders, due to cultural, legal, and religious restrictions.^33^ Overall, further research on familial cancer is needed in the Arab world, particularly large genetic screening programmes and improved genetic counselling.^34^ The Saudi Human Genome Project (SGHP) is an initiative that aims to describe the genetic distribution of cancer within Saudi Arabia, and this has been joined by programmes from Qatar, Egypt and the United Arab Emirates (Fig. 1). However, knowledge of targetable familial cancer genes in the Arab region remains sparse.

In this review, we discuss the prevalence and roles of familial and sporadic genetic mutations and their ethnic-specific differences, with a focus on the Arab world. Knowledge of this genetic landscape will be important for determining the prevalence of regional familial genetic predisposition to cancer.

### Epidemiology of familial cancer syndrome in the Arab world

Genetic cancer epidemiology represents the study of hereditary factors that are responsible for cancer initiation, metastasis, and prognosis.^9,35^ Knowledge of the regional genetic epidemiology of cancer can facilitate the development of suitable therapeutic treatments.^9^ Unfortunately, such studies in the Arab world have been lacking due to limited recognition that cancer health outcomes are influenced by genetic as well as social, economic and environmental, behaviours. However, progress has been made as using the American College of Medical Genetics and Genomics (ACMG) guidelines, Jastanahia et al. performed a multi-centre cross-sectional study on 1858 children with cancer in Saudi Arabia and found that 704 (40.4%) out of 1742 patients fulfilled criteria for hereditary cancer syndrome.^36^ Of these patients, consanguinity was reported in 629 (38%) of cases, with 50 (2.9%) first-degree, 535 (30.7%) second-degree, and 272 (15.6%) third-degree relatives afflicted. The data obtained in this study suggested that as many as 4/10 children with cancer in Saudi Arabia are afflicted with the hereditary form of the disease, due largely to consanguinity. This highlights the need for further genetic epidemiology testing across the Arab world.

Breast cancer is the most common malignancy amongst women in Arab countries, with 50% of cases presenting before the age of 50 years.^37,38^ From 2009 to 2012, El Saghir et al. assessed 250 Lebanese women with breast cancer, considered to be at high risk of carrying mutations in the BRCA1 or BRCA2 tumour suppressor genes, due to presentation of the disease at a young age and/or a positive family history of breast or ovarian cancer.^10^ The results showed that 14 of 250 patients (5.6%) carried deleterious *BRCA* mutations (7 *BRCA1*, 7 *BRCA2*) and 31 (12.4%) had variants of uncertain significance. These results were somewhat surprising and suggest that the prevalence of BRCA mutations is lower than predicted in Arab women.^10^ The notion that *BRCA* mutations alone cause the high incidence of breast cancer in young Arab women was not supported. Given that the rates of developing breast cancer vary amongst other racial and ethnic groups, and carriers vary by region, there is an urgent need for ethnic-specific genetic programmes.

More recently, Younes et al. performed a systematic review to estimate the genetic epidemiology of ovarian cancer in 22 Arab countries. These ranged from a low incidence of 0.9/100,000/year in Saudi Arabia to a high incidence of 8.0/100,000/year in Sudan.^9^ The total number of ovarian cancer patients identified were 802, of which 53 harboured mutations in *BRCA1/2* genes. *BRCA1* mutations were more frequent than *BRCA2* mutations and eight of the identified mutations were unique to Arab populations. This highlighted the importance of *BRCA1/2* mutations for the high prevalence of ovarian cancer observed across the Arab world.^9^ This was further supported by Siraj et al. who characterised the spectrum of *BRCA1/2* mutations regarding prevalence and founder effects in Arab regions to advance genetic counselling.^39^ Despite clear progress in our understanding of ethnic-specific hereditary cancer, problems still remain that are specific to the Arab region. A major barrier to genetic epidemiology is the avoidance of cancer screening and lack of knowledge of the importance of cancer, its risk factors, and the benefits that could be gained through screening. Al Abdouli et al. examined the public’s understanding of colorectal cancer in the United Arab Emirates (UAE) in which when surveyed, 64% believed that colorectal cancer is uncommon, and 67% had no knowledge of colorectal cancer screening tests.^40^ This highlights the need for changes in cancer awareness, attitudes and practices across the UAE. Racial stigmas also present a barrier to genetic screening programmes in the Arab world. The diagnosis of breast cancer in the middle-east often occurs at a later stage and in a higher proportion of women aged 30–40. It has been reported that 90.7% of women are aware of breast cancer, but only 7.6% have basic knowledge of breast cancer screening activities. The major reason for these low numbers is that Arab society is fundamentally conservative, and Arab women rely on mothers and sisters for disease support and protection. It was thus suggested that breast cancer detection efforts in Arab regions should include men so that they can encourage their female family members to participate in breast cancer screening activities. It is likely that national genetic screening campaigns will have similar problems in the region. Improved education and the communication of the benefits of early cancer detection should be pursued to improve familial genetic screening programmes across the Arab world.

### Cancer from the genetic perspective

Using advanced genetic methods, researchers have been able to determine the potency of gene expression and defective proteins, and the detection of novel cancer biomarkers in afflicted families. In addition, various studies have explored the epigenetic mechanisms and their relationship to the development, and progression of cancer. Whilst many aspects of epigenetic regulation remain unknown, identification of pivotal genes allows a comprehensive map for further efforts to reduce heritable cancer in future generations. This is particularly important for genetic counselling and the inheritance of genes that predispose individuals to cancer.^41,42^ Herein, from the brain to the colon, we review our genetic knowledge of specific cancers, and ethnic genetic predispositions.

### Paediatric cancer

Approximately 10% of all new cancer diagnoses are due to inherited genetic traits.^43^ Individuals with specific germline mutations possess a higher likelihood of developing life-threatening cancers, typically at a young age. The genetic basis of numerous childhood, and adolescent cancers, and cancers in young adults have been reported and identified. The detection of mutations is critical for the lifelong management of these patients and can dictate genetic counselling, surveillance, and early therapeutic interventions.^44–50^ Although the prevalence of childhood cancer is rare, 100,000 children younger than 15 years of age die from cancer each year, and whilst a further understanding of the scale of the problem is required amongst Arab countries, the proportions are thought to be higher in developing countries.

Germline/inherited mutations can be either dominant or recessive. Similarly, the disease profiles of inherited cancer syndrome drastically vary, leading to both early-onset and late disease-onset, and marked variations in cancer presentation. The majority of cancer predisposition genes (up to 90%) are through the inhibition of tumour suppressors. Approximately 10% of cancer predisposition genes (including *ALK*, *KIT*, and *MET*) are gain-of-function mutations. Notable examples of known cancer syndromes and the genes involved include X-linked recessive Simpson Golabi Behmel syndrome (caused by *GPC3* mutations), X-linked lymphoproliferative disease (caused by SH2D1A abnormalities), autosomal recessive ataxia telangiectasia (*ATM* mutations), Bloom syndrome (*BLM* mutations), Fanconi anemiam (*FANC* family), MUTYH-associated polyposis (*MUTYH*), Nijmegen breakage syndrome (*NBN*), Rothmund-Thomson syndrome (*RECQL4*) and Werners syndrome (*WRN*) (reviewed in the ref. ^43^). Notable autosomal dominant syndromes include Hereditary breast-ovarian cancer syndrome (*BRCA1*/2 mutations), hereditary diffuse gastric cancer (CDH1 mutations), Howel-Evans syndrome (*RHBDF2*), Li-Fraumeni syndrome (*TP53* mutations), Lynch syndrome and constitutional mismatch repair deficiency syndrome (*MLH1, MSH2, MSH6, PMS2* mutations), neurofibromatosis type 1/2 (*NF1/2* mutations), prostate cancer (*HPC1*, *BRCA1/2*), retinoblastoma (*RB1*), and tuberous sclerosis (*TSC1/2*). Examples of these cancers and their associated mutations will be discussed herein.

### Brain tumours

Neurofibromatoses leads to an increased likelihood of cancer development, particularly for peripheral nerve sheath tumours and gliomas. Type I the most common type is neurofibromatosis, is characterised by benign neurofibromas around the peripheral nerves. Neurofibromatosis type I is caused by autosomal dominant mutations in the *NF1* gene. NF1 regulates cell division through its ability to regulate RAS and PI3K activity. Approximately 50% of neurofibromatosis type I cases have a recognised family history of *NF1* mutations. Although rare, cases of hereditary neurofibromatosis type 1 have been reported in Arab children.

Glioblastomas are malignant brain tumours that develop from astrocytes. Most GBMs are not inherited and occur sporadically. However, glioblastomas can occur in those with neurofibromatosis type 1, Turcot syndrome (*APC, MLH1, PMS2* mutations) and Li Fraumeni syndrome (*TP53*). In each of these conditions, mutations are inherited in an autosomal dominant manner. According to the WHO classification of glioblastomas based on histopathological origin, it is categorised as a primary brain tumour of neuroepithelial, glial origin.^51^ When glioblastoma is ranked according to its clinical and prognostic significance, it is within the highest grade IV and is highly aggressive.^52^ Glioblastoma can be a result of progression from less malignant glial tumours (secondary type) or can occur de novo (primary).^53^ Paediatric glioblastoma is another subtype of this disease. Primary tumours are the most common.

Glioblastomas have an incidence rate of 3.19 per 100,000 persons in the US, with rates 2.0 times higher in Caucasians than African–Americans.^52^ The incidence of glioblastoma is lower in Asians and Native-American.^54^ The current treatment strategy for glioblastoma patients combines maximal surgical resection, followed by radiotherapy with concomitant and adjuvant temozolomide (TMZ).^55–57^ Less than 5% of newly diagnosed glioblastoma patients however survive longer than 5 years after diagnosis. It is clear that improved diagnostic and therapeutic strategies are required.

A family clustering of glioblastoma is recognised but relevant hereditary factors still remain elusive. Primary, secondary, and paediatric glioblastomas do however have known distinct sporadic genetic and epigenetic alterations. The most common mutations influence PI3K/AKT/mTOR, Ras/RAF/MAPK, and p53/Rb signalling pathways.^58–61^ These include mutations in *TP53, PTEN*, and *CDK4*.^62–68^ TP53 signalling is altered in 87% of glioblastomas, mostly affecting p53, murine double minute-2 (*MDM2*), *MDM4*, and cyclin-dependent kinase (*CDK*) *N2A* genes. Around 78% of glioblastomas have disruptions in RB signalling and frequently show alterations in *RB1, CDK4, CDK6, CCND2*, and the cyclin-dependent kinase inhibitor 2 (*CDKN2*) family.^63,67,69^ *RTK/RAS/PI3K* activation is observed in approximately 88% of tumours, typically affecting known hereditary (*NF1*) and non-hereditary (*PIK3R1*, and *PIK3CA*) genes.^70–72^

A problem of glioblastoma is its presentation as a heterogeneity of altered genetic pathways, evidenced by The Cancer Genome Atlas Research Network’s study classification based on gene expression profiles.^73^ Glioblastoma can be genetically typed^66^; Classical glioblastoma, with its highest survival rates, harbours no *TP53* mutations, but high rates of *EGFR* mutations. Mesenchymal glioblastoma has frequent mutations of *NF1*, *TP53*, and *PTEN*, and aggressive chemotherapy can increase survival. Proneural glioblastoma has the highest mutation rates typically in *TP53*, *PDGFRA*, and isocitrate dehydrogenase (*IDH*), usually afflicting young adults. The neural type is more common in older patients and is correlated with frequent mutations in *IDH1*. The identification of these genes has encouraged drug development efforts focused on these pathways with limited success.^74–77^ The heterogeneous nature of the disease suggests that multiple approaches may be more effective.

Understanding the genetics and epigenetics of glioblastoma can distinguish various subgroups, often histologically indistinguishable. This could lead to the development of genetic glioblastoma classification with clinical impact, subgroup-specific treatment regimens, and improved design of future clinical trials.^78^ Recently, large population-based studies in the US revealed a 13% decreased risk in Hispanics compared with white glioblastoma patients.^79^ Recent studies using data from 21,184 glioblastoma patients in adult Hispanics Americans also reported an increased survival for Hispanics compared with white non-Hispanics.^52^ From a hereditary perspective, is it unclear if the differences in survival among races are due to differences in genetic factors, environmental exposures, or differences in treatment? Wiencke and colleagues found that secondary glioblastomas have more *TP53* mutations that occur more frequently in Black and Asian patients.^80^ Conversely, Whites tend to harbour *EGFR* amplifications.^66^ As both secondary glioblastomas and *TP53* mutations have an improved prognosis and longer survival times compared to primary glioblastoma and *EGFR* amplification, this may explain why Blacks and Asians with glioblastoma live longer than Whites.

Glioblastoma in the Arab world is less well understood. To understand hereditary cancer in the region, Backes et al. performed exome sequencing in an Arab family in which both parents were healthy, whilst both children had glioblastoma.^81^ The study reported 85 homozygous non-synonymous single nucleotide polymorphisms (SNPs) in both siblings that were heterozygous in the parents, and thus represented potential hereditary glioblastoma genes. In addition to known glioblastoma genes including *ERBB2*, *PMS2*, or *CHI3L1*, over 50 genes new genes were identified. Of these, they identified an accumulation of effects that potentially increased the likelihood of glioblastoma in the siblings, including a clustering of multiple variants in single genes (*PTPRB, CROCC*), aggregation of genes that influence specific pathways (focal adhesion or ECM receptor interactions) and genomic proximity (chr22.q12.2, chr1.p36.33).^81^ The reported variants underlined the relevance of genetic predisposition and cancer development in this family.

Breast cancer is both complex and heterogeneous, encompassing many entities with variable clinical behaviours and biological features. High-throughput molecular methods have increased the ability to characterise the genetic landscape of breast cancer, some of which are now included in clinical practice. These include prognostic gene signatures for oestrogen receptor (ER)-positive and HER2-negative breast cancer patients, the assessment of HER2/neu status by FISH or IHC, OncoType Dx tests and MammaPrint assessments of 70 genes associated with tumour recurrence. Hereditary breast cancer is caused by germline mutations that occur in *BRCA1, BRCA2, TP53, CHEK2, PTEN, ATM*, and *PPM1D*.^82^ The discovery of these genes has permitted their classification into two groups, namely high-penetrance and low-penetrance that interact with other genes and/or environmental factors to cause disease. The two most common breast cancer genes are *BRCA1* and *BRCA2*, both of which are required for homologous DNA repair.^1,10,83,84^ A loss of heterozygosity and hereditary mutations in *BRCA1* or *BRCA2* increase chromosomal instability, thus increasing the cancer risk. Mutations in these genes also stimulate malignant transformation. Pathologically, familial breast cancers due to *BRCA1* mutations differ to those caused by *BRCA2* mutations and non-familial breast cancer. Understanding these pathological differences along with the genetic history of the patient are required to offer individualised treatment regimens.^85^

Germline mutations in *BRCA1* and *BRCA2* are responsible for 90% of hereditary breast cancer cases in the Western world and their mutation therefore represents the most important marker for the early detection of breast cancer.^86–88^ The landscape of breast cancer in the Arab world is somewhat different. Although it is known that breast cancer is on the rise (Fig. 1), its genetic epidemiology was less well understood. From a disease perspective, Arab patients with breast cancer have an advanced stage disease and a younger age of onset compared to Western countries. The Arab genome project pioneered by Saudi Arabia has been tasked with the discovery of new hereditary biomarkers for breast cancer in the region.^89^ This need for this was highlighted by Rahman and Zayed, who suggested that unlike Western populations, *BRCA1/2* mutations are not significantly involved in hereditary breast cancer in Bahrain, Kuwait, Oman, Qatar, Saudi Arabia, and the United Arab Emirates (UAE).^90^ In the Arab region, Al-Eitan et al. provided evidence that genetic variations in *MMP9*, *TOX3*, and *DAPK1* genes contribute to the development of breast cancer in the Jordanian population,^91^ whilst Alshawati et al. identified single-nucleotide polymorphisms in *TP53* and *MDM-2* that increase the risk of breast cancer in ethnic Arab populations.^12^ Karakas et al. also reported the prevalence of *PIK3CA* mutations and the SNP rs17849079 in Arab breast cancer patients.^2^

### Breast Cancer

For many years, the incomplete cataloguing of germline alterations in hereditary breast cancer cases led to a lack of consensus on those patients who should be tested. To overcome this issue, Siraj et al. designed the hereditary oncogenesis predisposition evaluation (HOPE) including genes with known association to breast cancer and other tumours in 1300 Arab cancer patients.^92^ Pathogenic or likely pathogenic alleles in genes other than BRCA2, ATM and PALB2 accounted for ~16.8% of mutation-positive breast cancers in which a family history was lacking in 63.7% of mutation-positive cases.^92^ This highlighted how germline mutations to breast cancer predisposition extend beyond the classic hereditary cancer genes. In similar studies, Crawford and colleagues assessed 300 high-risk women previously shown as negative for BRCA1/BRCA2 mutations, in which 26 women were found to harbour 28 pathogenic mutations in 19 sequenced genes. These included *ATM, CDH1, CHEK2*, and *RAD51D* in cases of bilateral breast cancer and *CHK2, MHS6* and *NBN* in those with ovarian cancer.^93^ Lee and colleagues similarly reported the occurrence of MSH2, PMS2, and CHEK2 in four hereditary breast and ovarian cancer syndrome patients with a family history of cancer.^94^ This highlights the importance of multi-gene panel testing as a follow-on test for those with incomplete testing. Armed with the knowledge that hereditary cancer is not strictly familial, this highlights the need for more widespread screening programmes in Arab regions.

Regarding ovarian cancer (OvCa) over 20% of ovarian tumours possess hereditary susceptibility. OvCa is common amongst Arabs with one of the highest global incidences. The reported numbers are likely to be higher due to underdiagnoses and underreporting. On the genetic level, up to 85% of hereditary OvCa cases are thought be caused by germline mutations in the *BRCA* genes. However, OvCa is not limited to BRCA susceptibility and other suppressor genes and oncogenes have been reported. RAD51 truncating mutations confer a reported 6-fold increased risk of OvCa but cause only a small increase in BCa susceptibility.^95^ PALB2 mutations (a BRCA2-interacting protein) have been also been reported in families negative for BRCA mutations with OvCa. Several groups have assessed the role of CHEK2 mutations in OvCa, particularly the missense variant I157T.^96^ This mutation was identified as involved in cystadenomas, borderline tumours, but not high-grade OvCa.^97^ Other variants including del1100C and A252G have also been reported but their association with OvCa is controversial. The Mre11 complex consists of Mre11, NBS1, and RAD50 and is a critical component of the DNA repair machinery. Three OvCa associated mutations have been reported including Mre11 913C>T (Arg305Trp), NBS1 448C>T (Leu150Phe), and RAD50 687delT (stop codon at 234).^98^ BARD1 germline mutations (c.1690C>T, p.Gln564X; c.1315- 2A>G; c.1977A>G) have also been reported as pathogenic for familial OvCa and BCa.^99,100^

### Colorectal cancer

Whilst most patients with Stage II/III colorectal cancer can be cured through combined surgery, radiotherapy and chemotherapy, treatment is costly and recurrence is frequent.^40^ There has been a remarkable improvement in our molecular understanding of colorectal cancer over the last three decades that has revolutionised numerous aspects of care. Lynch syndrome (LS) is the most common cause of hereditary colorectal cancer and its early detection provides an opportunity for preventive cancer approaches.^101^ Genetic mutations can make some tumours less responsive to treatment and the stratification of patients into genetic subgroups for targeted therapies represents an efficacious strategy to improve the clinical effects of treatment. It is now understood that genetic subsets of colorectal cancer carry different risk factors, disease prognoses, and responses to treatment.

In the case of inherited colorectal cancers, many are attributed to hereditary nonpolyposis (HNPCC), familial adenomatous polyposis (FAP) and other related but variable syndromes.^102,103^ Up to 30% of the patients fall into this category, with their first-line or second-line relatives having colorectal cancer. Novel and/or de novo germ-line mutations of the adenomatous polyposis coli (APC) occur in up to 25% of FAP patients when untreated, the incidence of colorectal cancer is high.^11,104–106^ The most frequent germ-line APC mutations occur on codons 1061 and 1309. Hereditary nonpolyposis is the product of mutations in the *MMR* genes, including *MLH1, MSH2, MSH6, PMS1*, and *PMS2*.^107–115^ Mutations in *MLH1* and *MSH2* are most common. Juvenile polyposis syndrome is caused by mutations in the bone morphogenetic protein receptor, type 1A (BMPR1A) or SMAD family member 4 (SMAD4), both of which are tumour suppressors.^116–123^ Cowden syndrome, results from mutations in *PTEN*. Homozygous mutations in the base excision repair (BER) pathway gene mutY DNA glycosylase (*MUTYH*) leads to MUTYH-associated polyposis syndrome, and heterozygous MUTYH mutations are observed in familial colorectal cancer

The molecular characterisation of colorectal cancer may help identify familial predispositions, permit the movement from conventional chemotherapy drugs to biomarker-driven treatments in advanced cases. Again, the identification of specific prognostic subgroups that can again be explored among the Arab population where known colorectal cancer drivers including *KRAS, NRAS, BRAF*, and *PIK3CA* mutations differ across populations. Al Shamsi et al. determined the mutational frequencies of these drivers in the Arab population^11^ in 198 cases (99 Arab patients and 99 Western patients). The frequency of *KRAS, NRAS, BRAF*, *TP53, APC*, and *PIK3CA* mutations were similar between Arab and Western populations, but *SMAD4* mutations were of lower frequency whilst *FBXW7* mutations were more frequent. Studies comparing Arab colorectal cancer occurrence to the Jewish population concluded that colorectal cancer is more advanced, aggressive and symptomatic in Arab populations.^124,125^ Since Arab patients are younger at the time of diagnosis, familial specific genetic variations are predicted to be involved. Armed with this information, screening amongst genetically high-risk groups in the Arab world in addition to policies designed to encourage healthier living are now required to reduce colorectal cancer incidence in the region.

### Prostate cancer

The incidence and mortality rates of prostate cancer show significant discrepancies among countries and ethnicities. Among people of European descent, prostate cancer is the most common cancer,^126^ but its prevalence is lower in men in the Middle East and North Africa.^127^ Prostate cancer is extremely heterogeneous compared to other tumours, and accordingly, various familial and sporadic mutations have been identified that increase its risk.^128,129^ Those with multiple single-gene polymorphisms and a family history of prostate cancer are at the highest risk.^126,128–132^ These include *BRCA1* and *BRCA2*, *MMR* mutations including *MLH1, MSH2* and *MSH6, PMS2, HOXB13*, checkpoint kinase 2 *CHEK2, NBN*, BRCA1-interacting protein C-terminal helicase 1 (*BRIP1*), and *ATM*.^129–131^ The potential of these findings is underscored by the recent approval of olaparib for *BRCA1/BRCA2* or *ATM*-mutated, metastatic, castrate-resistant prostate cancer. This highlights the impact of genetic testing in therapeutic strategies.^133–136^ It should be noted that, genetic profiling has not replaced PSA monitoring, prostate gland MRIs, or biopsies for screening. The presence of mutations associated with prostate cancer encourages patients to undergo earlier and more frequent screening with the hope of earlier diagnosis and treatment.

The molecular changes involved in prostate cancer have not been broadly explored across the Arab world.^127^ Prostate cancer is more frequent amongst men of African descent, which is attributed to ethnic-specific differences in genotype frequencies for both *SRD5A2* and *CYP3A4*.^137^ The CAG repeat length of the androgen receptor also differs according to population and is associated with prostate cancer.^138–141^ Progress in this area is required in the Arab world.

## Familial cancer genetic counselling clinic in Saudi Arabia

The worldwide demand for cancer genetic counselling is growing.^41^ Studies investigating cancer susceptibility within families have suggested genetic links to an array of malignancies at the population level.^14,142–144^ Genetic screening offers increased monitoring and surveillance of those with a risk of cancer, in addition to prophylactic, risk-reducing interventions.^144^ Fifteen relevant guidelines were developed to provide recommendations on genetic counselling (Supplementary Material) and were in general agreement of the importance of genetic counselling prior to *BRCA* testing, including breast cancer risk-reduction procedures including mastectomy and oophorectomy.^37^ Genetic counselling is a “process of helping people understand and adapt to the medical, psychological and familial implications of genetic contributions to disease”.^22,145–148^ Genetic counselling concerning cancer risks has benefited individuals and their relatives through improved adherence to risk management, increased knowledge of genetics, improved patient satisfaction, and cost.^149^ Conversely, negative outcomes, such as test result misinterpretation, incorrect medical management, and psychological distress can arise when genetic testing is performed in the absence of adequate genetic counselling.^150^ For example, many societies struggle to deal with cultural stigmas associated with harbouring a genetic disease, ultimately disadvantaging the afflicted families through social events including marriage refusal.^151^ This can lead to a refusal to participate in some regions.

Two of the first clinics in Saudi Arabia (both in Riyadh) that offered genetic counselling for familial cancer are King Fahad Medical City (KFMC) at the comprehensive cancer centre, and the Saudi Diagnostic Laboratory (SDL), Cancer Genetic Counselling clinic at King Faisal Specialist Hospital & Research Centre. Patients referred to this process must complete a minimum of two in-person visits (Fig. 1). At the initial visit, risk assessments are performed, and if applicable, informed consent for genetic testing is obtained. At the initial visit, a family history is obtained and the life risk for specific cancers is calculated. If a patient’s life risk is ≥20%, they are considered “high risk” and eligible for higher-level cancer screening processes, including more frequent mammograms. Furthermore, risk-reduction procedures (mastectomy, and oophorectomy) are also offered. For those who consent to genetic testing, a post-test visit is arranged for the interpretation of test results. During this visit, implications for other family members will are discussed, in addition to the patient’s future management of their familial cancer risk. Medications, surgeries, and lifestyle changes are discussed.

A challenge in familial cancer genetic counselling in Saudi Arabia is the pervasive belief that cancer is not genetic, as many attributes the aetiology of cancer to non-biological causes.^42^ A further challenge is navigating client fears regarding genetic counselling sessions.^23,152^ In Saudi Arabia, “’counselling” is not a term in general use and patients have no prior understanding of the process. When creating appointments, patients are asked an extensive panel of questions regarding their family history of cancer, which can cause distress. A further stress is that families receiving genetic counselling are provided negative information on a family member’s health.^50^ The decision to select the correct genetic test and how the data are interpreted and communicated the patient remain ongoing issues in these areas.^22,23,41,42^

An additional challenge is determining the goals of testing and the information required. Furthermore, genetic counsellors must successfully communicate risk perception to their patients.^143,153^ Genetic counsellors provide significant levels of long-term psychosocial support to patients and their families, termed “support counselling”.^154^ This allows genetic counsellors to help the patients cope with the test data.

### The state of the profession in the Middle East

Due to increased demand, the genetic counselling profession, led by Saudi Arabia, is expanding in the Middle East, and requires increased professionalism to accommodate future patients. Saudi Arabia established a master’s degree training programme in 2005,^155^ and the establishment of a professional society that focuses specifically on genetic counselling is planned. Active societies for medical genetics in the Middle East include the Saudi Society of Medical Genetics^155^ the National society of Human genetics in Egypt^156^ and the African Society of human genetics.^157^ Medical societies outside the middle-East have also been developed^158,159^

In the Middle East, the societies need to evolve to provide a distinct and coordinated forum for genetic counselling networking. The SSMG has proposed serves as an umbrella organisation and sponsors the formation of a Saudi-led genetic counsellor professional society. This society serves to integrate Middle-Eastern genetic counselling and could represent a nexus from which training and education can occur. To-date, those in the field of medical genetics know relatively little about genetic counselling. The development of professional societies can serve an educational role for both patients and colleagues.

Currently, Saudi Arabia is implementing a national strategy termed Vision 2030.^160^ This broad vision entails commercial and governmental goals, including improved public health and healthcare delivery, and the expansion of Saudi Arabia’s educational and research infrastructure.^161^ Organising and professionalizing the genetic counselling field in the Middle East is not only necessary to serve patients, but provides an opportunity for the Middle East to excel in this area globally.

## Conclusions

Familial cancer syndromes due to inherited mutations that increase the risk of tumour development account for ~5–10% of all malignancies and are generally characterised by early-onset cancers.^6^ In the majority of familial tumours, the risk is associated with monogenic hereditary disease. The ability to identify germline variants in familial cancer has been challenging due to incomplete cataloguing of cancer-mutations and disagreements on those who should be tested. What is clear is that before we can understand familial cancers, we must first identify relevant cancerous mutations that show prevalence in individual ethnicities. This is being increasingly recognised in the Arab world and our genetic understanding of cancer in the region is growing. This must now be combined with familial testing to define novel hereditary cancer drivers that can provide genetic counselling to families in the face of high consanguinity.

### Reporting summary

Further information on research design is available in the Nature Research Reporting Summary linked to this article.

## Supplementary information

Reporting Summary

## Data Availability

No datasets were generated or analyzed during the current study.
